# Tailoring Pectin-PLA Bilayer Film for Optimal Properties as a Food Pouch Material

**DOI:** 10.3390/polym16050712

**Published:** 2024-03-05

**Authors:** Nurul Saadah Said, Ibukunoluwa Fola Olawuyi, Won Young Lee

**Affiliations:** 1 School of Food Science and Technology, Kyungpook National University, Daegu 41566, Republic of Korea; nurulsaadah.said@gmail.com (N.S.S.); ifolawuyi@knu.ac.kr (I.F.O.); 2 Research Institute of Tailored Food Technology, Kyungpook National University, Daegu 41566, Republic of Korea

**Keywords:** pectin, PLA, bilayer film, biodegradable packaging, film optimization, food pouch

## Abstract

This study focuses on developing a biodegradable film using a novel hybrid citrus peel pectin. A bilayer approach with PLA was proposed and optimized using Response Surface Methodology (RSM) to complement pectin films’ mechanical and barrier property limitations. The optimized film composition (2.90 g PLA and 1.96 g pectin) showed enhanced mechanical strength with a tensile strength (TS) of 7.04 MPa and an elongation at break (EAB) of 462.63%. In addition, it demonstrated lower water vapor (1.45 × 10^−10^ g/msPa), oxygen (2.79 × 10^−7^ g/ms) permeability, and solubility (23.53%). Compared to single-layer pectin films, the optimized bilayer film had a 25% increased thickness, significantly improved water barrier (3806 times lower) and oxygen barrier (3.68 times lower) properties, and 22.38 times higher stretchability, attributed to hydrogen bond formation, as confirmed by FTIR analysis. The bilayer film, effectively protected against UV and visible light, could be a barrier against light-induced lipid oxidation. Moreover, it demonstrated superior seal efficiency, ensuring secure sealing in practical applications. The bilayer pouch containing mustard dressing exhibited stable sealing with no leakage after immersion in hot water and ethanol, making it suitable for secure food pouch packaging.

## 1. Introduction

The widespread use of plastics derived from non-renewable resources, especially those generated from petrochemicals, has raised concerns regarding environmental pollution and resource depletion [1]. Synthetic packaging materials, known for their non-biodegradable characteristics, have prompted a shift towards biodegradable packaging solutions sourced from renewable biopolymers such as polysaccharides (pectin and starch), lipids, and proteins (collagen and gelatin) [2,3,4,5,6].

Pectin, a renewable biopolymer with properties that contribute to cost-effectiveness, biodegradability, renewability, and abundance, holds promise as a raw material in the food industry [7]. In particular, novel pectin extracted from hybrid citrus by-products remains unexplored, presenting unique potential for new applications, especially in food packaging. Our previous studies found that Kanpei citrus is a promising material for packaging, showcasing superior mechanical and barrier properties compared to other hybrid citrus pectins [8]. However, despite these advantages, the commercial viability of Kanpei citrus pectin as a packaging material still needs to be improved by certain limitations, such as weak mechanical strength and barrier properties. To address these limitations, researchers have explored blending pectin with other biopolymers. The focus has been particularly on hydrophobic biodegradable polymers such as polylactic acid (PLA) to enhance mechanical and permeability characteristics, making them well-suited for diverse applications.

PLA is synthesized from lactic acid derived from renewable resources such as corn or other carbohydrate sources [9]. Additionally, PLA has been approved by the U.S. Food and Drug Administration (FDA) for its application in food-contact material [10]. It offers transparency, rigidity, and suitability for various fabrication processes, making it a promising material for food packaging [11]. However, blending PLA with pectin poses challenges, primarily because PLA is typically dissolved in chemical solvents such as acetone or chloroform, while pectin film development mainly occurs in a water-based solution. This difference in solvent compatibility leads to phase separation when the two components are combined, hindering effective mixing. Researchers have been exploring more effective strategies to enhance the synergistic mechanism between different polymer blends. Techniques such as layering, extrusion, and electrospinning have been employed to overcome challenges such as phase separation, aiming to unlock the potential of synergistic biodegradable materials suitable for various applications [12,13,14].

Bilayer or multilayer film packaging involves combining different materials in different layers to achieve specific properties [15]. Typically, a hydrophobic polymer such as PLA serves as the outer layer for moisture resistance and mechanical stability, while the inner layer may consist of a barrier material such as pectin to enhance oxygen barrier properties [16]. Adjusting each layer’s layering sequence and thickness allows for optimal performance in specific applications. The objective is to create a synergistic combination that maximizes each material’s strengths while minimizing its weaknesses. One approach to achieving optimal mechanical strength and barrier properties is optimization, and response surface methodology is suitable for this purpose.

To date, limited information is available on bilayer film composed of PLA and pectin-based polymers as materials for food pouches. Previous studies on pectin and PLA films utilized commercial pectin developed through extrusion processes and mixed as composite films [17,18,19]. In contrast, this study adopts the casting method for fabricating, optimizing, and characterizing bilayer film, with PLA as the outer layer and pectin-based material as the inner layer. This approach allows for a comprehensive assessment of the single and interaction factors influencing film properties, offering insights into the synergistic effects of the bilayer process. Furthermore, this study focuses on the potential application of pectin extracted from hybrid citrus peel, a novel and renewable source. The optimized bilayer film formulation will be compared to single pectin and PLA films to evaluate their efficiency as food packaging materials. Specifically, the bilayer film’s application as a food pouch for storing mustard dressing addresses the need for packaging solutions that provide adequate barrier properties and mechanical strength, catering to the requirements of the food packaging industry.

## 2. Materials and methods

### 2.1. Materials

Kanpei (KP), derived from the Citrus hybrid *Nishinokaori* × *Citrus reticulata Ponkan*, was obtained from a local farm on Jeju Island, Republic of Korea. The peels were sheared into fine pieces using a sharp-edged scissor. Subsequently, the sliced fragments were placed on steel trays and subjected to an 18 h drying process in a hot air oven set at 45 °C. The dried peels were blended and sifted through a 50-µm mesh sieve to obtain the powdered sample. The resulting powder was stored in a tightly sealed container under refrigerated conditions before experimental analysis. Polylactic acid (PLA) pellets were purchased from Shanghai Huiang Industrial Co., Ltd. (Shanghai, China), with a specific gravity ranging from 1.20 to 1.30 g/cm^3^, a melt flow index of 2–12 g/10 min at 190 °C/2.16 kg, a melting degree below 155 °C, and a glass transition temperature of 60 °C. Chemical solvents such as chloroform, obtained from Daejung Chemicals & Metals Co., Ltd. (Siheung-si, Gyeonggi-do, Republic of Korea), glycerol from Samchun Chemical Co., Ltd. (Seoul, Republic of Korea), and citric acid from Duksan Chemicals (Ansan, Republic of Korea), were of analytical grade.

### 2.2. Extraction of Pectin

Pectin extraction was conducted under acidic conditions, following the method outlined in our previous study [8] for pectin extracted from hybrid citrus peel. Citrus peel powder was dissolved in 0.1 M citric acid at a 1:30 (g/mL) ratio by adjusting the citric acid’s pH between 2.2 and 2.4. The mixture was homogenized at 5000 rpm for 1 min (Daihan Scientific Co., Ltd., Wonju, Gangwon-do, Republic of Korea) and heated in a water bath at 95 °C for 90 min. Subsequently, the solution underwent centrifugation (Supra-22K, Hanil Science Industrial, Incheon, Republic of Korea) at 6000 rpm for 20 min. The resulting supernatant was filtered through a filter bag, and 95% ethanol was added to the filtrate in a 2:1 ratio. The mixture was chilled at 4 °C for approximately 18 h to induce precipitation. The crude pectin was washed twice with 95% (*v*/*v*) ethanol following centrifugation at 6000 rpm for 10 min before being freeze-dried. The dried pectin was further processed by a blender and sieved through a 50 µm mesh sieve, resulting in a finely powdered sample.

### 2.3. Bilayer Film Preparation

Citrus pectin films were developed following the procedure outlined in Çavdaroğlu et al. [20], with a slight modification. Kanpei citrus peel pectin (amount specified in Table 1) was mixed with 100 mL of distilled water. The mixture was homogenized at 5000 rpm for 2 min using a homogenizer (Daihan Scientific Co., Ltd., Republic of Korea) before being left to stir in a water bath at 60 °C for 30 min. Next, 30% glycerol (*w*/*w*) was added, and the solution was continuously stirred at 60 °C for another 30 min. Glycerol was added as a plasticizer due to its stability and compatibility with pectin hydrophilic biopolymer chains [21]. To remove air bubbles, the mixture was vacuum degassed using an ultrasonic device (JAC-3010, Kodo Technical Research Co., Ltd., Hwaseong, Republic of Korea). After that, films were cast on leveled plastic plates using 25 mL of the film-forming solution for each plate and dried at 45 °C in an oven dryer for 2 days. After drying, the films were carefully removed from the plates and set aside for further processing.

For the pre-dry PLA component, the PLA pellet was pre-dried in an oven dryer (OTEC-004-m, Republic of Korea) at 60 °C overnight, and then a predetermined amount of PLA, as outlined in Table 1, was dissolved in 100 mL of chloroform through 2 h of stirring until complete dissolution was achieved. The subsequent step involved the combination of PLA and dried pectin films: 25 mL of the PLA solution was applied to a glass casting plate, onto which the dried citrus pectin film was delicately placed. The solution was allowed to dry naturally at room temperature until both PLA and pectin films were thoroughly dried and harmoniously fused.

### 2.4. Experimental Design Using Response Surface Methodology (RSM)

The design and optimization of a bilayer pectin film with PLA were designed using response surface methodology (RSM) based on central composite design (CCD). The factorial part, outlined in Table 1, constituted a design involving all possible combinations of factors at two levels (high, +1, and low, −1 levels). The center points (coded level 0), representing the midpoint between high and low levels, were repeated three times. This design comprised 13 runs, with pectin and PLA quantities as factors, and response variables such as tensile strength (TS), elongation at break (EAB), water vapor permeability (WVP), oxygen permeability (OP), and moisture content were measured. After the optimized validation formulation was finalized, the optimized bilayer pectin/PLA film was compared to single pectin and PLA films for their mechanical, water, and oxygen barrier properties, moisture content, water solubility, thickness, color, light transmittance, FTIR structure, heat seal properties, biodegradability rate, and application as a food pouch.

### 2.5. Mechanical Properties of Films

The tensile strength (TS) and elongation at break (EAB) of the films were determined using a QMESYS Universal Material Testing Machine (QM100S, 1.96 kN, Komachine, Yongin City, Gyeonggi-do, Republic of Korea) following the ASTM Standard Method D 882-02 [22]. The dried films were conditioned for 24 h at a controlled temperature of 25 °C before testing to ensure consistent measurements. The films were then precisely cut into strips measuring 6 mm in length and 10 mm in width. The gauge distance was set at 20 mm during the testing process, and the crosshead speed was maintained at 10 mm/min. The thickness of the films was measured using a digital micrometer (NR 293-244-30, Mitutoyo, Kawasaki, Japan) to ensure precision. The collected data, including TS (MPa) and EAB (%), were calculated using the QM-Pro Software (RS232 version).

### 2.6. Water Vapor Permeability (WVP) of Films

The water vapor permeability (WVP) of the films was assessed through a modified version of the ASTM E96 technique [23]. The films were employed to seal open-top vials containing calcium chloride pellets. The initial weight of the vial and film was recorded before placing them in a desiccator with distilled water at room temperature. Over a six-day period, the vials were weighed every 24 h. The average thickness of the film was measured using a digital micrometer (NR 293-244-30, Mitutoyo, Kawasaki, Japan). The WVP was then determined using the following formula [Equation (1)]:

WVP (g/msPa) = Δw × x/At × (ΔP)(1)

where Δw is the change in weight of the cup containing the sample film (g), x is the average thickness of the film (m), A is the permeation surface area (m^2^), t is the time elapsed, and ΔP is the difference in partial pressure of the atmosphere (2339 Pa).

### 2.7. Oxygen Permeability (OP) of Films

The oxygen permeability (OP) of the films was assessed using an adsorption approach based on the iron oxidation mechanism proposed by Zhang et al. [24]. A deoxidizing agent weighing 1 g, composed of NaCl, activated carbon, and reduced iron powder in a precise ratio of 1.5:1.0:0.5, was prepared before adding it to an open-top vial sealed with the films. After the initial weighing, the vials were transferred to a desiccator filled with a saturated barium chloride solution (90%) and kept at 25 °C for 2 days. The OP rate of the vials was determined at 24 h intervals using the following [Equation (2)]:

OP = ∆M × x/A × t(2)

where ∆M represents the change in mass of the vial (g), x is the film thickness (m), A is the exposed area of the film (m^2^), and t (s) is the equilibrium time.

### 2.8. Moisture Content and Water Solubility (WS) of Films

The composite films (50 × 20 mm) were initially weighed (W0) and dried at 105 °C until a consistent weight was obtained (W1). The film’s moisture content (MC) was estimated using the formula from Said et al. [8], as shown in [Equation (3)]:

(3) $$\mathrm{MC}=\frac{W1-W0}{W0}\times 100$$

Subsequently, the dried films were submerged in distilled water at room temperature for 24 h to assess water solubility (WS). The undissolved films were dried again at 105 °C until a constant weight was reached (W2), and the water solubility was calculated as in [Equation (4)]:

(4) $$\mathrm{WS}=\frac{W1-W2}{W2}\times 100$$

### 2.9. Thickness, Color, and Light Transmittance of Films

The films’ thickness and color characteristics were measured using a digital micrometer gauge (NR 293-244-30, Mitutoyo, Kawasaki, Japan) and a Chroma Meter colorimeter (CR-300, Minolta Co., Osaka, Japan), respectively [8,25]. Film samples (50 × 20 mm) were employed for both measurements, with the thickness determined by averaging values from five different points on each sample to ensure accuracy. A color analysis was performed prior to calibration for the colorimeter with a white reference plate (CR-300: L* = 93.80, a* = 0.3130, b* = 0.3191) to ensure precise color measurements. The parameters L* (lightness), a* (red-green), and b* (yellow-blue) were recorded. For light transmittance analysis, the UV-Vis spectrophotometer (Shimadzu Co., UV-2550, Tokyo, Japan) was employed to assess light transmission within the wavelength range of 200–800 nm to measure the film barrier properties against UV and visible light as well as the opacity [26]. Film strips (1 × 4 cm) were positioned directly in the measuring cell, with the blank cuvette used for the baseline reading. Opacity was determined by calculating absorbance units (A) at 600 nm readings, divided by thickness units (D, mm), using the formula as specified in [Equation (5)].

(5) $$\mathrm{Opacity}\;({\mathrm{Amm}}^{-1})=\frac{A600}{D}$$

### 2.10. Structural Analysis of Films

The Attenuated total reflection-Fourier transform infrared (ATR-FTIR) spectra of the films were obtained using an FTIR spectrometer (Frontier, Billerica, MA, USA). Each film was scanned at a resolution of 4 cm^−1^ within the range of 4000 to 400 cm^−1^ [27].

### 2.11. Heal Seal Properties of Films

Seal strength and seal efficiency were determined according to the method described by Nilsuwan et al. [28]. Film samples (2 × 4 cm) were cut into strips, and two strips of bilayer film were heat-sealed together at 150 °C for 5 s using an impulse sealer. The sealed samples were conditioned at 25 °C for 24 h before testing. Peel strength and seal efficiency were measured using a QMESYS Universal Material Testing Machine (QM100S, 1.96 kN, Komachine, Yongin City, Gyeonggi-do, Republic of Korea) at 25 °C. The samples underwent tensile loading at 100 N until seal failure occurred. Seal strength [Equation (6)] and seal efficiency [Equation (7)] were calculated using the following formulas:

Seal strength (N/m) = Peak force/Film width(6)

Seal efficiency (%) = (Peak force/Tensile force) × 100(7)

### 2.12. Biodegradation Rate of Films

Film biodegradability was evaluated by desiccating film pieces (2 × 2 cm) in a desiccator with silica gel until a consistent weight was reached [25]. These desiccated film pieces were then buried in 100 g of soil and exposed to sunlight for aerobic degradation. Degradation rates were measured at 0, 3, 7, and 14-day intervals by weighing each sample. After removing the soil, the samples were weighed until a constant weight was achieved. The weight loss of the film was calculated using [Equation (8)].

(8) $$\mathrm{Weight}\;\mathrm{loss}\;(\%)=\frac{W1-W2}{W1}\;\times 100$$

where W1 is the initial weight and W2 is the final weight.

### 2.13. Evaluation of Biodegradable Pouches for Packaging as Sauce Dressing Pouch

This method was adapted from the procedure outlined by Janjarasskul et al. [29], with a slight modification. Mustard dressing was repackaged into bilayer pouches, sealed on all sides, maintaining a surface area similar to the original small mustard product pouch in the market. Subsequently, the bilayer film was immersed and stirred in hot water (90 °C) and ethanol for 2 h to assess any leakage compared to pectin and PLA single films.

### 2.14. Statistical Analysis

The RSM Design-Expert 6.0.10 software (Stat-Ease 2003) was utilized to optimize independent variable conditions, providing model summary statistics, ANOVA, R^2^, Adj-R^2^, CV, and AP to evaluate the validity and fit of the polynomial model equation. Statistical significance between samples was determined using SPSS version 20.0 through one-way ANOVA, followed by the Tukey post-hoc test (*p* < 0.05).

## 3. Results and Discussions

### 3.1. Optimization of Bilayer Film Consisted of Pectin and PLA

#### 3.1.1. Model Development

The levels of factors (PLA (X1), pectin (X2)) and the effect of their interaction on tensile strength (TS, Y1), elongation at break (EAB, Y2), water vapor permeability (WVP, Y3), oxygen permeability (OP, Y4), and solubility (Y5) were determined through RSM-CCD. Thirteen experimental runs were conducted at different factor-level combinations, as specified in Table 2. The model F values were significant (*p* < 0.05) for all responses, indicating the significance of all model terms. Simultaneously, the lack-of-fit test provided insights into the adequacy of the fitted model, assessing the error arising from any deficiency in the model [30]. The lack-of-fit *p*-values for all responses were non-significant (*p* > 0.05), indicating that the model is both fit and adequate for explaining the data. The mathematical equations representing the response surface model for all responses were derived using the actual values of regression coefficients. These equations are expressed as follows:

Final equation in terms of coded factors:

(9) $$\mathrm{TS},\;Y_{1}=+6.76+0.44X_{1}+1.00X_{2}+0.75X_{1}^{2}-2.87X_{2}^{2}+0.21X_{1}X_{2}-0.25X_{1}^{2}X_{2}-1.31X_{1}X_{2}^{2}$$

(10) $$\mathrm{EAB},\;Y_{2}=+428.38+249.55X_{1}+132.54X_{2}-89.33X_{1}^{2}-168.82X_{2}^{2}+16.84X_{1}X_{2}-142.03X_{1}^{2}X_{2}-174.53X_{1}X_{2}^{2}$$

(11) $$\mathrm{WVP},\;Y_{3}=+1.547\times 10^{-10}+3.139\times 10^{-11}X1_{\;}+7.203\times 10^{-11}X_{2}+3.151\times 10^{-11}X_{1}^{2}+6.228\times 10^{-11}X_{2}^{2}\;-9.753\times 10^{-11}X_{1}X2_{\;}+4.152\times 10^{-11}X_{1}^{2}X_{2}-1.11\times 10^{-10}X_{1}X_{2}^{2}$$

(12) $$\mathrm{OP},\;Y_{4}=+9.619\times 10^{-8}+3.055\times 10^{-8}X_{1}+1.247\;\times \;10^{-8}X_{2}\;+3.578\times 10^{-8}X_{1}^{2}+1.640\times 10^{-8}X_{2}^{2}-7.626\times 10^{8}X_{1}X2_{\;}+3.955\times 8X_{1}^{2}X_{2}-6.926\times 10^{-8}X_{1}X_{2}^{2}$$

(13) $$\mathrm{Solubility},\;Y_{5}=+26.82-10.13X_{1}+7.14X_{2}\;-0.73X_{1}^{2}+8.35X_{2}^{2}+11.54X_{1}X_{2}\;+3.45X_{1}^{2}X_{2}\;+12.80X_{1}X_{2}^{2}$$

Moreover, the model effectively explains response variations, as evident from the satisfactory coefficients of determination (R^2^), adjusted coefficients of determination (Adj-R^2^), coefficients of variation (CV), and adequate precision (AP) values presented in Table 3. The R^2^ values for responses Y1, Y2, Y3, Y4, and Y5 are 0.9710, 0.9914, 0.9783, 0.9265, and 0.9858, respectively (as shown in Table 3). High R^2^ coefficients indicate strong correlations between experimental and predicted response values. Therefore, the model reliably predicted the film’s mechanical properties, WVP, OP, and solubility. However, a model with a high R^2^ value may provide poor predictions for new observations or estimates of mean responses.

Some regression models consider adjusted R^2^, as its value does not increase with adding more variables [31]. R^2^ assumes that each variable explains the variation in the dependent variable, while adjusted R^2^ measures the percentage of variation based on independent variables that affect the dependent variable. In Table 3, adjusted R^2^ values for Y1, Y2, Y3, Y4, and Y5 are 0.9304, 0.9794, 0.9478, 0.8237, and 0.9660, respectively. The CV values range between 6.91 and 12.94, reflecting the standard deviation as a percentage of the mean. Adequacy precision (AP), indicating model discrimination, is desirable above a ratio of 4. In this model, AP ratios range from 11.755 to 27.342, indicating an adequate signal [30]. Therefore, based on the analysis, these models can be employed to explore the design space for enhancing film properties.

#### 3.1.2. RSM Tensile Strength (TS)

**Figure 1:**
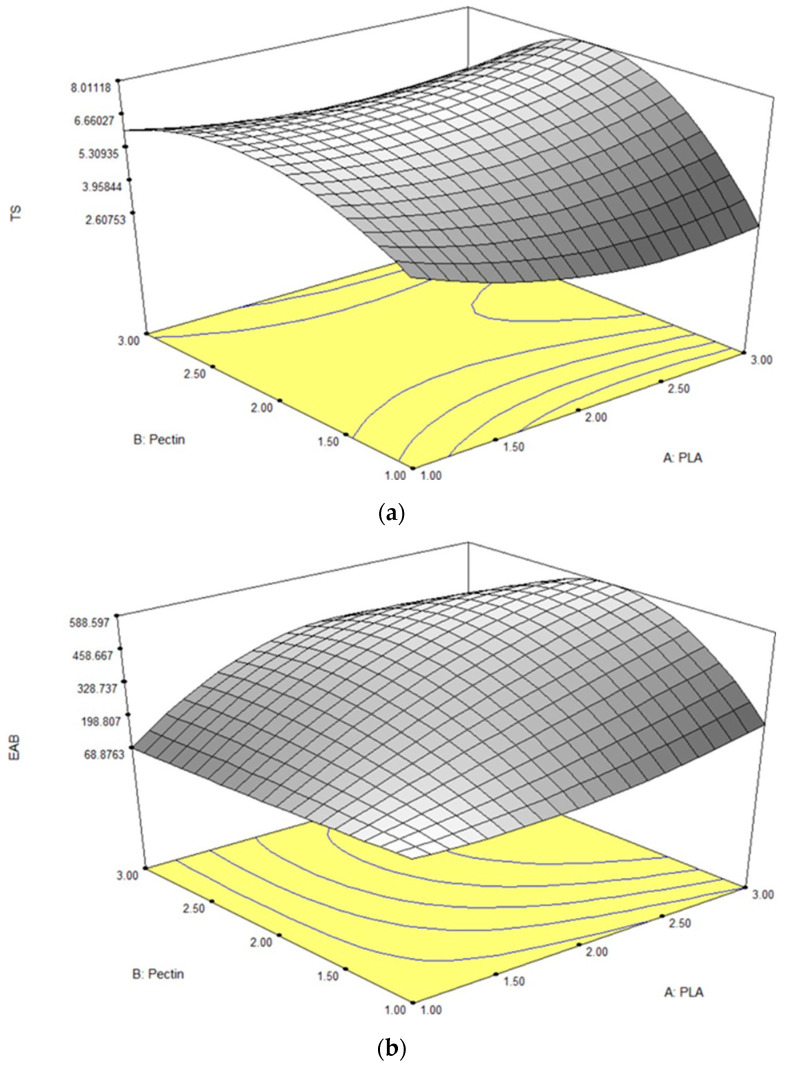

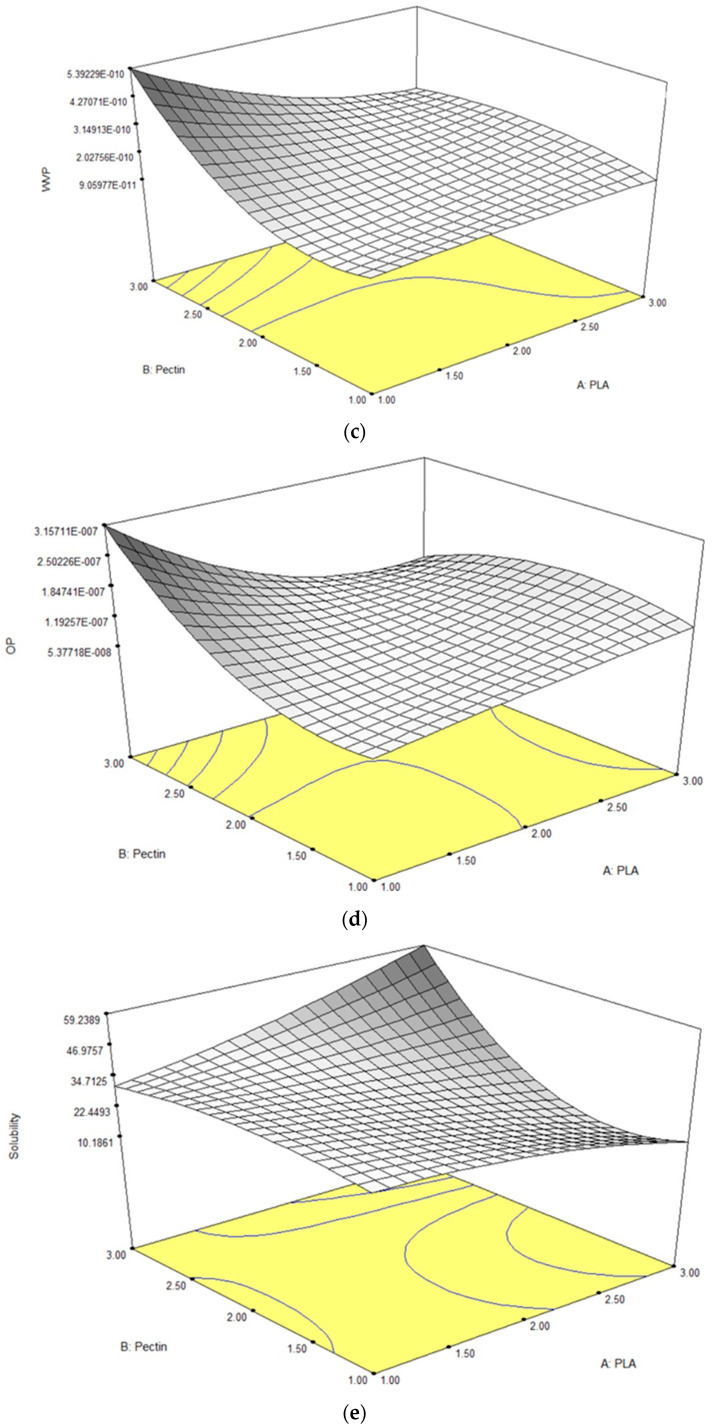
Response surface plots for (**a**) TS, (**b**) EAB, (**c**) WVP, (**d**) OP, and (**e**) solubility in bilayer film as influenced by different concentrations of pectin and PLA.

The tensile strength (TS) reflects a material’s ability to withstand stress before deformation or breakage [32]. In this study, the pectin/PLA bilayer film demonstrated TS values ranging from 2.83 to 7.89 MPa, as detailed in Table 2. A response surface plot revealed that higher TS levels were associated with increased PLA content and an intermediate level of pectin inclusion (Figure 1a). Analysis of individual parameters indicated that linear and quadratic correlations with pectin significantly influenced TS (*p* < 0.05). However, the interaction between pectin and PLA did not significantly impact TS (*p* > 0.05), as shown in Table 3. Equation (9) highlighted that the linear term of both PLA and pectin contributed to the film’s TS. The coefficient magnitude for pectin (1.00) exceeded that of PLA (0.44), indicating a more substantial effect on TS. Interestingly, negative coefficients in the quadratic term of pectin’s influence suggested an optimal concentration range for maximizing TS. Deviation beyond this range in either direction decreased TS, supported by the 3D contour graph illustrating a decline in TS when pectin concentration exceeded the optimal point above 1.96 g (*w*/*v*).

Modifying the pectin and PLA proportions significantly influences the TS of the bilayer film. Adjusting these levels can potentially change the film’s strength characteristics. Higher PLA content contributes to a more uniform and aligned structure, thereby enhancing overall TS [33,34]. A moderate amount of pectin facilitates better interlocking between PLA molecules, reinforcing the film’s strength. This elasticity complements PLA’s strength, producing a balanced, strong, and somewhat flexible bilayer film. The findings were consistent with optimization studies involving PLA and acetylated starch films, where increased substitution degree and PLA concentration improved the films’ TS [33]. However, the interaction between pectin and PLA may not significantly affect TS due to their differing molecular arrangements and properties, leading to a lack of synergistic enhancement in the film’s TS [35].

#### 3.1.3. RSM Elongation at Break (EAB)

EAB, representing the ratio of changed length to the initial length after breakage, ranged from 73.55% to 579.25%. ANOVA results in Table 3 indicated significant effects (*p* < 0.05) of linear and quadratic terms for both PLA and pectin, including their quadratic interactions, on EAB. Both linear and interaction terms positively impacted the film’s EAB based on Equation (10). In the linear term, PLA had a higher magnitude (249.55) than pectin (132.54), emphasizing PLA’s pronounced impact on elasticity. However, in the quadratic term, both PLA and pectin had negative coefficients, indicating that deviations from optimal levels could negatively affect film elasticity. Pectin had a more substantial negative impact (−168.82) compared to PLA (−89.33), suggesting that extreme levels of either can reduce the film’s elasticity, as illustrated in the 3-D contour plot (Figure 1b).

The significant impacts of PLA and pectin on EAB can be explained by their individual properties. High PLA content increases stiffness, potentially causing a higher change in length upon breaking. An intermediate level of pectin enhances overall flexibility, allowing for a more significant change in length upon breakage [35]. The absence of a significant interaction effect between PLA and pectin suggests their combined influence on EAB is not synergistic, as their individual effects may already be dominant. Hence, this shows that more than the inclusion of PLA is needed to provide excellent stretchability and ductility to the film. With the aid of pectin, similar findings were noted in the bilayer film of gelatin and PLA, which exhibited lower tensile strength (TS) but higher elongation at break (EAB) compared to other films [11]. The changes in the bilayer film structure were attributed to the formation of hydrogen bonds between pectin and PLA during the layering process.

#### 3.1.4. RSM Water Vapor Permeability (WVP)

The WVP test for the films is a widely used measurement to evaluate their ability to limit moisture transfer between food and the surrounding environment, thus impacting food shelf life [36]. A lower WVP value indicates better moisture protection, which is crucial for extending the shelf life of food products [37]. In this study, the WVP values ranged from 1.18 to 5.40 × 10^−10^ g/msPa, as detailed in Table 2. ANOVA results (Table 3) revealed that the linear and quadratic effects of pectin alone significantly influenced WVP (*p* < 0.05). Additionally, the interaction effect between PLA and pectin significantly impacted WVP values (*p* < 0.05). In the context of Equation (11), the positive sign in both the linear and quadratic terms for the pectin variable indicates that increasing pectin content leads to an elevation in the film’s water vapor permeability (WVP). Notably, pectin’s higher magnitude compared to PLA across linear and quadratic terms emphasizes its stronger influence on promoting higher WVP. This can be visualized in the steeper curvature of a 3-D contour plot inclined towards a higher concentration of pectin, as shown in Figure 1c. 

Pectin’s hydrophilic nature can explain the lower WVP values with less pectin inclusion. In this study, the use of homogalacturonan (HG)-type pectin extracted from Kanpei hybrid citrus, characterized by its linear structure, led to larger pores in the film matrix, resulting in higher moisture absorption and weaker hydrophobic properties compared to highly branched pectin-based films [8,38]. A higher OH composition in HG pectin could also increase the film’s affinity for water vapor [39]. These water molecules can then penetrate into the films through the voids between pectin molecular chains, leading to higher permeability [35]. As for the interaction effect between PLA and pectin, it likely arises from their combined influence on the film’s structure. When PLA and pectin interact, molecular arrangement and packing within the film’s matrix can be influenced. This arrangement can create convoluted paths for water vapor molecules, requiring them to navigate a complex route through the film, thereby slowing their diffusion [40]. Moreover, denser molecular packing and higher thickness can restrict the unhindered movement of water vapor molecules, ultimately leading to a decrease in WVP values [41]. Thus, as observed in the significant interaction result, this combined effect reduces WVP values, signifying enhanced moisture protection, an essential aspect of food packaging material.

#### 3.1.5. RSM Oxygen Permeability (OP)

In film packaging testing, OP measures how easily oxygen molecules pass through the material. A lower OP value signifies better prevention of oxygen passage, which is crucial for preserving product quality by minimizing processes such as oxidation and spoilage [42]. The results showed that the OP responses were observed between 5.17 × 10^−8^ to 3.20 × 10^−7^ g/ms. ANOVA results, as shown in Table 3, revealed that only the interaction term of PLA and pectin had a significant effect (*p* < 0.05) in the film’s OP, while the linear and quadratic terms for those two variables showed no significant effect (*p* > 0.05). Referring to Equation (12), both the linear and quadratic terms for each variable displayed a positive sign, with PLA exhibiting larger magnitudes (3.055 × 10^−8^ and 3.578 × 10^−8^, respectively) than pectin (1.247 × 10^−8^ and 1.640 × 10^−8^, respectively). This aligns with the equation’s pattern, as is visually evident in Figure 1d, where the plotted curve showcases a larger curvature leaning towards higher concentrations of PLA and a steep curvature in the highest pectin concentration. 

It is recommended to minimize OP throughout the film matrix passage. The negative interaction in Equation (12) suggests a synergistic effect between pectin and PLA, in addition to the only significant effect (*p* < 0.05) observed in the interaction term in Table 3, emphasizing that combining these two components is crucial for achieving lower OP compared to using them individually. The higher crystallinity of both pectin and PLA nature characteristics is mentioned as one of the factors significantly impacting the lower permeation of oxygen gases throughout the film matrix [43,44]. Interactions such as hydrogen bonding and van der Waals forces between pectin and PLA as bilayer film [45] also play a pivotal role in strengthening the film structure, optimizing it for a more effective barrier against oxygen permeation. Therefore, this demonstrates that the development of bilayer films, as opposed to single PLA or pectin films, is essential for providing enhanced protection against oxidation. This attribute prolongs the shelf life of intended food products, making it favorable as a material for food pouches.

#### 3.1.6. RSM Solubility

The film solubility test evaluates the dissolution or dispersion of a film in a specific solvent, typically water, which is crucial for assessing water resistance in applications such as food packaging and pharmaceuticals [46,47]. Films with lower solubility exhibit increased stability, preventing dissolution or disintegration when exposed to moisture, thus maintaining structural integrity over time for effective protection of packaged products. In this study, solubility values ranged from 14.74% to 42.81%, as detailed in Table 2. ANOVA results (Table 3) demonstrated that both linear, quadratic, and interaction effects of pectin and PLA significantly influenced solubility (*p* < 0.05). In Equation (13), the positive sign in the linear and quadratic terms for pectin indicates that higher pectin content leads to increased film solubility, illustrated by a steeper curvature in the 3-D contour plot towards higher pectin concentrations (Figure 1e).

The explanation could be that pectin, being a hydrophilic polysaccharide, has an affinity for water molecules [48]. Higher pectin concentrations contribute to a more porous and water-absorptive film matrix, increasing solubility [38]. The positive coefficient for pectin indicates that, on its own, higher pectin concentration tends to increase solubility by enhancing the water-absorbing properties of the film. In combination with PLA, the positive interaction term (+11.54) indicates a synergistic effect, fostering a film matrix that preserves structural integrity while enabling water penetration, ultimately enhancing solubility. Thus, although the combination may not achieve lower solubility, it contributes to the creation of a more robust and flexible film.

#### 3.1.7. Numerical Optimization

Numerical optimization was employed to determine the optimal levels of independent variables for achieving desirable values across all responses. The optimum conditions were identified at 2.90 g of PLA and 1.96 g of pectin, as outlined in Table S1. Under these recommended conditions, the corresponding predicted values for TS, EAB, WVP, OP, and solubility were 6.76 MPa, 428.38%, 1.55 × 10^−10^ g/msPa, 9.62 × 10^−8^ g/ms, and 26.83%, respectively.

#### 3.1.8. Validation Test

Validation tests were conducted to assess the actual values of TS, EAB, WVP, OP, and solubility under the optimized conditions (2.90 g of PLA and 1.96 g of pectin). Results in Table S2 revealed that TS, EAB, and OP values exceeded the predicted values, reaching 7.04 MPa, 462.63%, and 2.79 × 10^−7^ g/ms, respectively. Conversely, WVP and solubility were lower (1.45 × 10^−10^ g/msPa and 23.53%) than the predicted values. While EAB and solubility fell outside the 95% confidence interval, all other responses remained within this interval, indicating a 95% confidence that the average data falls within the specified range. Additionally, all responses were within the 95% prediction interval, which is wider due to added uncertainty and scattered data in predicting individual responses. In summary, the optimized PLA/pectin-based bilayer film exhibits favorable characteristics for food packaging applications, offering improved strength and flexibility while demonstrating lower permeability and solubility. This combination creates an optimal material condition for effective food packaging. In the next application, optimized bilayer film was further evaluated in pouch form and compared to single pectin and PLA film.

### 3.2. Characterization of Optimized Bilayers against Single Pectin and PLA Films

#### 3.2.1. Physical Properties of Optimized Film

**Figure 2:**
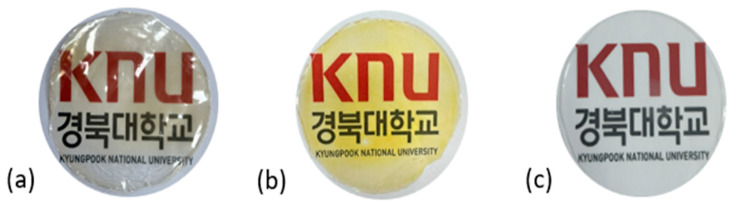
The appearance of (**a**) optimized bilayer pectin/PLA film, (**b**) single pectin film, and (**c**) single PLA film.

The illustration of all films is depicted in Figure 2. The thickness analysis revealed that the optimized bilayer film (0.229 mm) had a higher value compared to single pectin (0.058 mm) and PLA (0.069 mm) films, likely due to increased polymeric material inclusion and molecular weight, resulting in a more intricate and thicker structure, which corresponds with observations from other studies [49,50,51]. In food pouch applications, a higher film thickness is generally preferred for superior mechanical strength, enhanced barrier properties, increased protection for products with sharp edges, contributing to overall pouch rigidity, and maintaining food quality under longer storage times [52,53].

Regarding color attributes as shown in Table 4, the L* value for the optimized bilayer film was 94.47, indicating a lighter appearance compared to pectin (88.20) and PLA (92.11). The positive a* value (1.09) suggests a shift towards redness in the bilayer film, while pectin film displayed a negative value (−6.01), and PLA exhibited a value of 0.23. In terms of yellowness (b*), the pectin showed a considerably higher positive value (29.42) associated with enhanced yellowness compared to others.

The solubility of a film serves as a critical indicator of its resistance to a solvent, particularly in the context of active food packaging materials [54]. Table 4 illustrates the water solubility characteristics across different film types, showing that pectin-based film exhibits significantly higher water solubility (64.77%) compared to PLA and bilayer pectin/PLA film samples (2.22% and 20.53%, respectively). The higher water solubility in pectin films extracted from the Kanpei citrus variety is attributed to elongated GalA chains (68.31%) containing carboxyl groups [8]. These groups readily engage in hydrogen bonding with water molecules. Conversely, the hydrophobic nature of PLA, characterized by recurring lactic acid units, imparts reduced susceptibility to water absorption and solubility [55,56]. This study’s objective is to improve the application of pectin as a film material, and this improvement is evidenced by the optimized bilayer film’s substantial reduction in water solubility, which is nearly three times lower than that observed in the single pectin film. The combination of PLA creating intramolecular bonds with pectin, limiting the availability of free hydroxyl groups, aligns with the observed reduction in water solubility. A similar finding was reported by Chaichi et al. [57], where hydrogen bonding interactions between nanocellulose and pectin matrix led to fewer free pectin molecules connected to water molecules. This observation aligns with the statement that a decrease in hydroxyl groups, resulting from strong molecular interactions such as hydrogen bonding or dipole-dipole interactions, may lead to reduced water solubility [58]. Hence, the reduction of water solubility in bilayer film confirmed the films’ stability in aqueous environments, making them suitable for packaging applications in various forms.

In the analysis of moisture content (Table 4), it is observed that the bilayer film has a higher moisture level (16.86%) compared to the individual pectin and PLA films (13.50% and 7.00%, respectively). While PLA does not dissolve in water, its water-repellent nature and high moisture vapor transmission rates suggest its ability to absorb moisture from its surroundings. This property contributes to the increased moisture content in bilayer films with pectin and PLA, exceeding films made solely of pectin or PLA. The fact that pectin is water-attracting enhances the moisture absorption of the bilayer film, leading to its higher moisture content [59]. Therefore, combining pectin and PLA in a bilayer film results in a higher moisture content due to pectin’s water-attracting quality and PLA’s ability to absorb moisture. Even though the bilayer film has a higher moisture content, this characteristic could enhance flexibility, which is beneficial in particular packaging and biomedical applications.

#### 3.2.2. Barrier Properties of Optimized Film

##### Water Vapor Permeability Properties (WVP)

WVP is a crucial parameter in food packaging, indicating a film’s ability to resist moisture. Low WVP values signify effective protection against water transmission, safeguarding foods from external environmental moisture [60]. The optimized bilayer film achieved the lowest WVP at 0.002 × 10^−7^ g/msPa, showing a substantial 412 to 3800-fold reduction compared to individual pectin and PLA films within the range of 0.825−7.612 × 10^−7^ g/msPa (Table 4), probably attributed to the extended diffusion path created by the bilayer structure and the tight intermolecular interactions within the film [59]. The study highlighted the synergistic effect of combining pectin and PLA, emphasizing their pivotal role in enhancing the water vapor barrier properties of the films. 

However, pectin/PLA bilayer film has resulted in higher WVP compared to starch/PLA bilayer film (0.21−0.42 × 10^−10^ g/msPa) [61] and soy protein isolate/PLA (2.30−3.40 × 10^−11^ g/msPa) [62] bilayer film. This could be attributed to the hydrophilic nature of the pectin used in this study, which further facilitates higher WVP. A study by Mehraj and Sistla [63] emphasized that pectin films with a higher degree of esterification (DE) demonstrated a greater presence of polar hydroxyl groups. In this study, the pectin used had a DE of 93.34%, accompanied by a methoxyl content within the range of 66.33% [8]. The water transport mechanism in polymer films typically involves the absorption of water molecules and subsequent migration facilitated by polar hydroxyl groups. Hence, higher WVP was observed to align with the expected behavior of hydrophilic groups in polymer films. However, in accordance with this study’s objectives, the bilayer pectin/PLA film exhibited a lower WVP than individual pectin and PLA films, enhancing its potential as a superior food packaging material.

##### Oxygen Permeability Properties (OP)

Gas permeability, a steady-state parameter, describes the extent and rate of gas (e.g., O_2_ and CO_2_) dissolution and passage through packaging films [34]. Thus, aside from opting for low water permeability across film material, maintaining a storage atmosphere with low O_2_ concentrations is also crucial to prevent oxidation and microbial spoilage in food [64]. Oxygen permeability showed that optimized pectin/PLA film has a significantly lower value (0.022 × 10^−5^ g/ms) compared to PLA (18.107 g/ms) with a near 823-fold reduction (Table 4). Interestingly, pectin film also resulted in around 220 times lower OP than PLA film as it possessed a complex structure and branching of pectin molecules. The pectin used in this study was extracted from Kanpei hybrid citrus, which had a high composition of RG-I (24.18%), including 1.64% rhamnose, 9.37% galactose, and 11.52% arabinose [8]. The elongated chain of galacturonic acid (68.31%) coupled with these branching side sugars may create a more tortuous path for oxygen molecules, resulting in lower permeability. Moreover, it has been noted that the hydrophilic nature of polysaccharide-based films, such as pectin, renders them water-sensitive and offers an excellent barrier against non-polar permeants such as oxygen and aroma compounds [65].

The optimized pectin/PLA bilayer film exhibited lower oxygen permeability (OP) compared to individual films, aligning with previous studies. The bilayer chitosan/pectin film (0.70–0.75 g/ms) exhibited lower OP compared to chitosan single film (1.39 g/ms) and various types of single pectin films (0.95–1.14 g/ms), attributed to the polar interaction during polyelectrolyte assembly, forming a dense two-layer film matrix with effective intermolecular bonding [59,66]. This helps to fill the voids between the film matrix and creates a more tortuous transport path that hinders oxygen flow [66].

##### Light Permeability Properties

UV light can cause gloss and softness loss, color changes, and harm sensitive substances such as foods and drugs, as its energy can break down chemical bonds such as C-C, C-O, and C-H [67]. Table S3 presents the UV and visible light transmission data for films within the 200–800 nm wavelength range. No significant differences (*p* > 0.05) were observed between optimized bilayer film and pectin films in the UV range (200–400 nm), indicating similar UV light barrier properties. The UV absorbance of the PLA film resulted in a lower value than the other two films. A film with high absorbance values serves as effective packaging, providing coverage and protection against UV light [68,69]. This emphasizes pectin’s superior UV-blocking abilities over standalone PLA film, as pectin’s effective absorption in the 340 nm UV light range is facilitated by the carbonyl group’s participation in electronic transitions from non-bonding *(n*) to anti-bonding pi (π*) orbitals, typically associated with UV light absorption [70]. As for the visible light range (400–800 nm), optimized bilayer film had significantly higher absorbance compared to single pectin and PLA film. Pectin film, however, resulted in lower absorbance in the visible light range compared to both PLA and optimized bilayer film. This observation is consistent with previous findings highlighting the characteristic of pectin, which exhibits weak absorption for visible light and strong absorption for UV light [70]. The reason for pectin’s low absorbance in the visible light range is attributed to its molecular composition and structure, which may include properties that minimize light absorption within that wavelength spectrum.

As for the light transmission characteristics, the opacity values in the optimized pectin/PLA film were significantly higher (*p* < 0.05) compared to the single films (Table S3), indicating lower transparency in the single films. This increase in opacity may be associated with the observed higher thickness in the optimized bilayer film, contributing to reduced transparency. Additionally, the crosslinking reactions among the film matrix might lead to the formation of a denser polymeric matrix, resulting in reduced transparency of the film material [25]. The findings in this study were in accordance with previous results, showing increased opacity in the bilayer film due to interactions within the film matrix, as observed by Chakravartul et al. [71] and Liu et al. [72] for pectin bilayer film. Higher opacity can be advantageous in applications where blocking light is crucial, such as in packaging materials for protecting light-sensitive contents. This aligns with the study’s aim to produce an optimized film suitable for food pouches. In summary, the results suggest that the optimized pectin/PLA films effectively act as barriers against light-induced lipid oxidation.

#### 3.2.3. Structural Analysis (FTIR)

**Figure 3:**
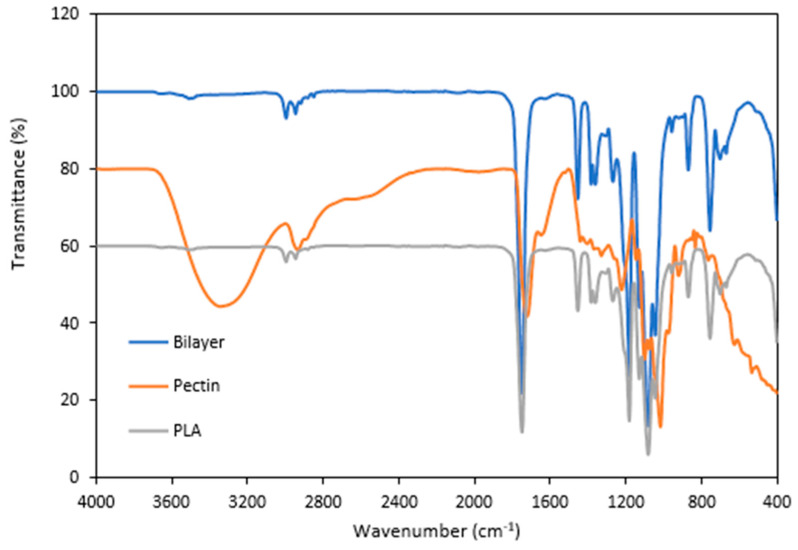
FTIR analysis of optimized bilayer film as a comparison to the single film layer.

The functional groups of the optimized pectin/PLA bilayer film were analyzed by infrared spectroscopy to identify whether new bonds were created between the pectin and PLA layers. Figure 3 shows the FTIR spectra of single PLA, single pectin film, and bilayer pectin/PLA film. The PLA, pectin, and PLA/pectin films had five characteristic absorption peaks. In the FTIR analysis of a single pectin film, the broad band in the 3600–3200 cm^−1^ region indicates O-H stretching due to hydrogen bonding facilitated by GalA. The sharp bands around 3000–2800 cm^−1^ are attributed to C-H absorption, representing pectin CH, CH_2_, and CH_3_ vibrations induced by GalA methyl esters. The O-CH_3_ stretching band, indicating pectin methoxylation, is obscured by the significant O-H stretching response in the 3670–2500 cm^−1^ range. Prominent bands at 1700–1820 cm^−1^ suggest the presence of methyl-esterified carboxyl groups (COO-R). This aligns with the structural features of pectin from hybrid citrus Kanpei, as reported in the literature by [8] through FTIR analysis. While the FTIR analysis of PLA films revealed another distinctive absorption peak indicative of its molecular structure, the bands in the range of 3000−2900 cm^−1^ signify the C-H stretching of methyl groups and the O-H vibration of carboxylic acid [35,61,73]. The peak observed at 1748 cm^−1^ corresponds to the C=O stretching vibration of carbonyl groups [62], while deformations of -CH_3_CH stretching are observed around 1452 cm^−1^ and 1360 cm^−1^ [73]. Additionally, the region of 1200 − 1000 cm^−1^ signifies C-O stretching [73].

For optimized bilayer film structural analysis, a distinct peak at 3508 cm^−1^ was observed, indicating the presence of OH groups, which were absent in PLA. The O-CH_3_ stretching band, indicating pectin methoxylation, was remained by a significant O-H stretching response in the broad region of 3670–2500 cm^−1^ [74]. In addition, the presence of prominent bands at 1700–1820 cm^−1^ suggested the existence of methyl-esterified carboxyl groups (COO-R), indicating the main pectin structure remained unchanged in the bilayer film [8]. However, the peak at 1630–1600 cm^−1^, representing carboxylate group [COO⁻] stretching in the pectin film, was not observed in the bilayer film. Additionally, configurations in the 625 to 640 cm^−1^ range, specific to hybrid citrus pectin, were also absent in the bilayer film [8,75]. The layering of pectin with PLA may lead to alterations in the structure and molecular interactions, affecting certain functional groups’ vibrational modes, as observed in the spectrum. The most important observation to note is the structural difference observed from the difference in peak intensity between the bilayer structure and single film, particularly at 1748 cm^−1^ and 1081 cm^−1^. The reduction in the carbonyl stretching band around 1700–1750 cm^−1^, associated with the C=O group in the FTIR spectrum of the bilayer film compared to the single film, can be attributed to the formation of hydrogen bonds between functional groups [76] in pectin and PLA, including hydroxyl (OH), carboxyl (COOH), and methoxyl groups.

#### 3.2.4. Mechanical Properties

**Figure 4:**
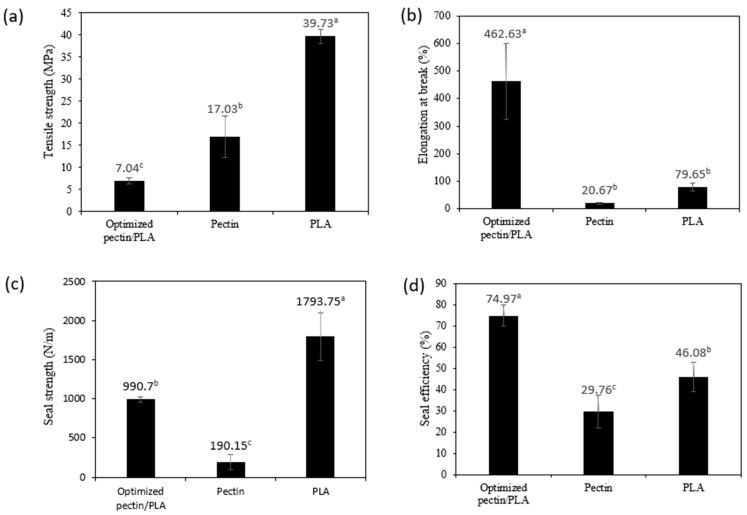
(**a**) Tensile strength, (**b**) elongation at break, (**c**) seal strength, and (**d**) seal efficiency of optimized bilayer film as a comparison to the single film layer. Different letters (^a–c^) indicate a significant difference (*p* < 0.05).

Film mechanical properties are crucial for maintaining integrity and enduring external stress during the processing, transporting, handling, and storing of packaged materials [35]. Figure 4 presents the mechanical properties, including tensile strength (TS) and elongation at break (EAB), of the optimized bilayer film (pectin/PLA) in comparison to the single pectin and PLA films. The TS of the optimized bilayer film is significantly lower at 7.04 MPa compared to pectin (17.03 MPa) and PLA (39.73 MPa). This phenomenon might be attributed to the reduction in TS in the optimized pectin film, which could be influenced by the tear force between the layers during stretching. Specifically, the outer PLA layer, responsible for bearing the external tensile load, may experience different mechanical behavior compared to the inner pectin layer, which could potentially break apart first. This differential response between layers contributes to the overall observed lower TS in the bilayer film [77]. This is supported by multiple studies that have observed a decrease in TS in bilayer films, as seen in cases such as PLA/gelatin, agar/chitosan, and PVA/agar [77,78]. The explanation for the higher TS observed in a single film of PLA compared to pectin can be explained by the molecular structure of PLA, which forms robust crystalline regions, providing greater strength [17]. In contrast, pectin’s natural polymer nature may lead to lower TS due to its amorphous structure and the presence of functional groups such as carboxylic, methyl ester, and methyl amide groups, potentially resulting in weaker intermolecular forces and mechanical strength [8,79].

Elongation at break (EAB) signifies the maximum length change a film undergoes before breaking in relation to its initial length, reflecting on the film’s flexibility and stretchability [80]. The results showed that even though the optimized bilayer film had the lowest resistance to break, it was significantly higher in stretchability (462.63%) than those of the other films, which is 5–22 times higher than the single films (PLA: 79.65% and pectin: 20.67%) (Table 4). Similar findings were noted in the bilayer film of gelatin and PLA, which exhibited lower TS but higher EAB than other films [28]. Compared to single films, the observed changes in bilayer film structure can be attributed to the formation of hydrogen bonds between pectin and PLA during the layering process. Differences in structure were evidenced by shifts in the intensity of peaks at 1748 cm^−1^ and 1081 cm^−1^, which indicated hydrogen bond formation as analyzed in FTIR bands (Figure 4). In the FTIR spectrum, the carbonyl stretching band, typically found around 1700–1750 cm^−1^, associated with the C=O group, may exhibit broadening or weakening due to the formation of hydrogen bonds between functional groups such as hydroxyl (OH), carboxyl (COOH), and methoxyl groups in pectin and carbonyl (C=O) in PLA. Similar observations were noted in the FTIR analysis of bilayer film by Wang et al. [76] that the formation of hydrogen bonds occurred based on the shift intensity of peaks at 1748 cm^−1^ and 1081 cm^−1^. Hence, this indicates that the optimized bilayer film has significant stretchability and strong durability to withstand heavier food loads, making it suitable as a material for food pouch applications.

#### 3.2.5. Heat Seal Analysis

Seal strength is a crucial factor in assessing the overall quality of packaging materials. The sealing capacity is a key indicator of its ability to effectively secure the contents inside the package, preventing any leakage during storage or transportation. In this study, the seal strength measures the force holding the sealed films together, while seal efficiency expresses the utilization of the film’s tensile strength in the heat-sealed area [28]. The results showed that PLA film exhibited significantly greater seal strength (1793.75 N/m) compared to both optimized bilayer (990.70 N/m) and pectin film (190.15 N/m), as shown in Figure 4. However, in terms of seal efficiency, the optimized bilayer film demonstrated the highest percentage (74.97%) compared to pectin (29.76%) and PLA (46.08%). The higher seal strength of PLA film compared to optimized bilayer and pectin film suggests that PLA alone has a stronger bonding ability during sealing. However, when considering seal efficiency, the optimized bilayer film utilizes its strength more effectively in the sealed area, resulting in a higher percentage of strength used for sealing compared to pectin and PLA films. The improved efficiency of the optimized bilayer film during sealing may be linked to the abundant OH in the bilayer film matrix, facilitating easy fusion during heating. This phenomenon aligns with findings in a study on heat-sealing properties between PLA and chitosan composite films conducted by Ye et al. [81]. In addition, compared to other films, the strong adhesion properties of pectin-PLA films are attributed to the amorphous and low crystallinity nature of PLA, which also contains low molecular weight oligomers. These characteristics enhance its polarity and chemical affinity with pectin at the interface, leading to favorable interlayer adhesion and stability over time. This was explained in the seal strength analysis conducted between bilayer films of starch and PLA by Hernández-García et al. [82]. Therefore, this demonstrates that the optimized formulation of a bilayer film comprising pectin and PLA can offer excellent heat strength, making it well-suited for packaging materials, particularly for food pouches that require secure sealing.

#### 3.2.6. Biodegradability Test

Table S4 presents the degradation results of films, depicting average weight loss values against degradation time for all samples. This illustrates the relationship between weight loss and degradation time over 14 days. During the 3-day observation period, the pectin film displayed complete degradation and was no longer recoverable. In contrast, PLA exhibited a minimal degradation rate of 1.89–6.82% over two weeks, primarily due to its polymer composition. PLA is expected to undergo complete biodegradation within approximately ten months through ester bond hydrolysis in the polymer backbone, influenced by factors such as temperature, pH, enzymes, and moisture [83]. In Table S4, the optimized bilayer film showed a loss of its yellow color, linked to the inclusion of the pectin layer, with over half of it degrading into the soil. The biodegradation rate increased from 38.69% at three days up to 71.32% after two weeks, suggesting that pectin played a significant role in the overall weight loss. Pectin, being a naturally occurring polysaccharide, serves as an energy source for most microorganisms, contributing to the accelerated biodegradation rate. The findings align with the biodegradation analysis of PLA in bilayer film with other polymers, indicating minimal degradation in the PLA layer and substantial degradation in other layered polymers such as protein or polysaccharide polymers [62,73]. Hence, this indicates the biodegradable potential of the optimized bilayer film, demonstrating its ability to naturally decompose in soil without causing harmful effects on land contamination or contributing to landfill problems.

#### 3.2.7. Stability and Leakage Test of Films as Food Pouch

This test is designed to simulate conditions that the pouch may encounter during use, including exposure to hot liquids and various environmental factors. The inclusion of ethanol in the test serves a crucial purpose by replicating the effects of alcoholic or alcoholic-containing substances that the pouch might come into contact with in real-world scenarios, especially during sanitizing processes. The bilayer pouch, containing mustard dressing, exhibited remarkable stability with no signs of leakage after 2 h of immersion in hot water and ethanol, as shown in Table 5. The bilayer film demonstrated superior performance compared to the individual pectin and PLA films, which exhibited signs of leakage after both treatments. It also demonstrated no penetration of an external solution into the pouch, and the film structure remained intact and rigid throughout the test. This contrasts with the pectin film, which exhibited a destroyed film structure, and the PLA film, which showed penetration of an outside solution into the pouch. The results indicated that both barrier and mechanical properties were successfully enhanced by optimizing the bilayer film process compared to single films. This underscores the enhanced integrity and performance of the bilayer film under conditions reflective of its intended use, especially as a biodegradable food pouch. For instance, the bilayer film designed as a dressing pouch can be seamlessly integrated with instant or packaged foods that require dispensing in small quantities. This design ensures convenient and controlled access to the dressing sauce, enhancing the overall user experience and practicality, especially in scenarios where portion control or on-the-go applications are essential.

## 4. Conclusions

In conclusion, we have successfully developed pectin and PLA bilayer film composed of PLA (2.90 g) and pectin (1.96 g), which exhibit enhanced film properties and versatility as a packaging material. The subsequent analysis of the optimized bilayer film, including physicochemical, structural, heat seal strength, biodegradability, and food pouch application for mustard dressing, revealed superior performance compared to individual pectin and PLA films. The bilayer film exhibited a thicker layer structure and lighter color, attributed to increased polymer material inclusion. The incorporation of PLA with pectin facilitated intramolecular bonding, limiting free hydroxyl group availability and contributing to the observed reduction in water solubility. Despite higher moisture content, the bilayer film demonstrated improved flexibility, evident by a significantly higher elongation at break (EAB) value, about 5–22 times higher than single films. However, the difference in response between layers during stretching contributed to lower tensile strength (TS) in the bilayer composite film. Nevertheless, optimized bilayer films achieved remarkable reductions in water vapor permeability (WVP) and oxygen permeability (OP), showcasing improvements over individual pectin and PLA films. The bilayer structure, with a complex matrix of diffusion paths and tight intermolecular interactions, contributed to the observed reductions in both WVP and OP. Notably, the optimized bilayer film demonstrated excellent UV and visible light transmission, serving as effective barriers against light-induced lipid oxidation. In terms of sealing efficiency, the optimized bilayer film demonstrated superior sealing properties, attributed to the abundant OH groups in the bilayer film matrix, which facilitated easy fusion during heating. Furthermore, the bilayer film demonstrated promising biodegradable potential, as evidenced by its ability to naturally decompose in soil. The stability exhibited by the bilayer pouch containing mustard dressing, with no signs of leakage after immersion in hot water and ethanol, further highlighted the film’s enhanced integrity and performance under conditions reflective of its intended use, especially as a biodegradable food pouch. Hence, through all these constructive analyses, it is evident that the optimized bilayer film formulation can generate superior film properties and functionality compared to individual films, particularly as a material for food pouch packaging.

## Figures and Tables

**Table 1:** Experimental domain of face-centered Central Composite Design (CCD).

Independent Variables	Unit	Symbol		Coded Variables Levels		
		Uncodified	Codified	−1 (α)	0	+1 (α)
PLA	g (*w*/*v*)	X_1_	x_1_	1.00	2.00	3.00
Pectin	g (*w*/*v*)	X_2_	x_2_	1.00	2.00	3.00

**Table 2:** Central Composite Design (CCD) employed for formulation of pectin/PLA bilayer film.

Design Point	Independent Variables		Dependent Variables				
	X_1_	X_2_	TS (MPa)	EAB (%)	WVP (g/msPa)	OP (g/ms)	Solubility (%)
1	−1	−1	5.00	126.20	1.18 × 10^−10^	6.30 × 10^−8^	32.48
2	0	0	6.29	468.25	1.29 × 10^−10^	9.54 × 10^−8^	23.76
3	0	0	6.56	437.03	1.75 × 10^−10^	5.17 × 10^−8^	29.16
4	0	0	6.54	417.60	1.45 × 10^−10^	1.07 × 10^−7^	28.15
5	−1	+1	6.08	73.55	5.40 × 10^−10^	3.20 × 10^−7^	30.56
6	0	+1	4.83	382.75	2.88 × 10^−10^	1.17 × 10^−7^	42.81
7	+1	0	7.89	579.25	2.17 × 10^−10^	1.55 × 10^−7^	16.46
8	+1	−1	2.85	242.58	1.53 × 10^−10^	1.37 × 10^−7^	14.74
9	+1	+1	4.78	257.28	1.85 × 10^−10^	8.89 × 10^−8^	58.99
10	0	0	7.39	435.25	1.31 × 10^−10^	1.32 × 10^−7^	24.84
11	−1	0	7.00	80.15	1.54 × 10^−10^	9.38 × 10^−8^	36.72
12	0	−1	2.83	117.68	1.44 × 10^−10^	9.25 × 10^−8^	28.53
13	0	0	7.20	402.48	1.94 × 10^−10^	1.10 × 10^−7^	27.19

TS = tensile strength; EAB = elongation at break; WVP = water vapor permeability; OP = oxygen permeability.

**Table 3:** ANOVA study for model fitting of tensile strength (TS), elongation at break (EAB), water vapor permeability (WVP), oxygen permeability (OP), and solubility of pectin/PLA bilayer film.

Source	TS				EAB				WVP				OP				Solubility			
	Sum of Squares	DF	F Value	*p* Value	Sum of Squares	DF	F Value	*p* Value	Sum of Squares	DF	F Value	*p* Value	Sum of Squares	DF	F Value	*p* Value	Sum of Squares	DF	F Value	*p* Value
Model	30.93	7	23.92	0.0015	338,677	7	82.40	<0.0001	1.479 × 10^−19^	7	321.6	0.0007	4.882 × 10^−14^	7	9.01	0.0139	1528.70	7	49.76	0.0003
Linear																				
A	0.39	1	2.14	0.2036	124,550	1	212.13	<0.0001	1.970 × 10^−21^	1	3.00	0.1439	1.867 × 10^−15^	1	2.41	0.1811	205.21	1	46.76	0.0010
B	2.00	1	10.38	0.0217	35,132.38	1	59.84	0.0006	1.038 × 10^−20^	1	15.79	0.0106	3.110 × 10^−16^	1	0.40	0.5540	101.93	1	23.23	0.0048
Quadratic																				
A^2^	1.54	1	8.35	0.0342	22,041.37	1	37.54	0.0017	2.742 × 10^−21^	1	4.17	0.0965	3.536 × 10^−15^	1	4.57	0.0856	1.46	1	0.33	0.5887
B^2^	22.72	1	123.03	0.0001	78,715.86	1	134.07	<0.0001	1.071 × 10^−20^	1	16.30	0.0099	7.428 × 10^−16^	1	0.96	0.3723	192.56	1	43.88	0.0012
Interaction																				
AB	0.18	1	0.98	0.3679	1134.01	1	1.93	0.2233	3.805 × 10^−20^	1	57.91	0.0006	2.326 × 10^−14^	1	30.05	0.0028	532.90	1	121.43	0.0001
Quadratic interaction																				
A^2^B	0.082	1	0.45	0.3679	26,894.80	1	1.93	0.0011	2.298 × 10^−21^	1	3.50	0.1204	2.085 × 10^−15^	1	2.69	0.1616	15.83	1	3.61	0.1160
AB^2^	2.28	1	12.34	0.0170	40,611.97	1	69.17	0.0004	1.643 × 10^−20^	1	25.01	0.0041	6.463 × 10^−15^	1	8.35	0.0342	218.46	1	49.78	0.0009
Residual	0.92	5			6101.63	5			3.285 × 10^−21^	5			3.870 × 10^−15^	5			21.94	5		
Lack of fit	0.028	1	0.12	0.7431	506.77	1	0.83	0.4126	1.840 × 10^−24^	1	2.242 × 10^−3^	0.9645	3.367 × 10^−16^	1	0.38	0.5704	1.44	1	0.28	0.6243
Pure error	0.90	4			2428.89	4			3.283 × 10^−21^	4			3.533 × 10^−15^	4			20.50	4		
Cor total	31.85	12			341,612	12			1.512 × 10^−19^	12			5.269 × 10^−14^	12			1550.64	12		
R^2^	0.9710				0.9914				0.9783				0.9265				0.9858			
Adj-R^2^	0.9304				0.9794				0.9478				0.8237				0.9660			
CV(%)	7.43				7.84				12.94				23.13				6.91			
AP	15.246				27.342				20.995				11.755				26.928			

**Table 4:** Characteristics of optimized bilayer film as a comparison to the single film layer.

Test	Optimized Bilayer Pectin/PLA Film	Pectin Film	PLA Film
	
Thickness (mm)	0.229 ± 0.01 ^a^	0.058 ± 0.00 ^b^	0.069 ± 0.00 ^b^
L*	94.47 ± 0.53 ^a^	88.20 ± 0.05 ^c^	92.11 ± 0.69 ^b^
a*	1.09 ± 0.04 ^a^	−6.01 ± 0.02 ^c^	0.23 ± 0.02 ^b^
b*	−2.92 ± 0.39 ^c^	29.42 ± 0.05 ^a^	−0.09 ± 0.02 ^b^
Solubility (%)	20.53 ± 0.22 ^b^	64.77 ± 2.50 ^a^	2.22 ± 0.03 ^c^
Moisture content (%)	16.86 ± 0.43 ^a^	13.50 ± 1.35 ^b^	7.00 ± 0.31 ^c^
WVP × 10^−7^ (g/msPa)	0.002 ± 0.00 ^b^	7.612 ± 1.03 ^a^	0.825 ± 0.03 ^b^
OP × 10^−5^ (g/ms)	0.022 ± 0.00 ^b^	0.081 ± 0.00 ^b^	18.107 ± 1.31 ^a^

WVP = water vapor permeability; OP = oxygen permeability. Different letters (^a–c^) indicate a significant difference (*p* < 0.05).

**Table 5:** Stability and leakage test of films as food pouches.

Visual Observation	Optimized Bilayer Pectin/PLA Film	Pectin Film	PLA Film
Original state	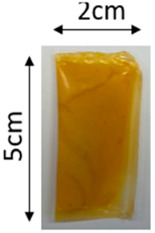	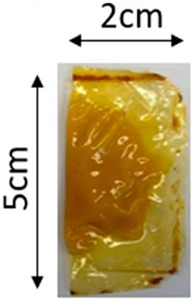	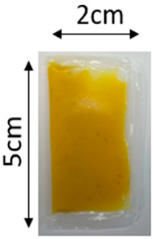
After treatment, the state of the water bath (90 °C for 2 h) and 90% ethanol solution (2 h)	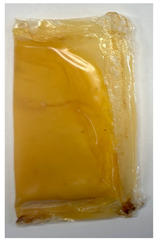 No leakage or penetration of an external solution into the pouch was observed, and the film structure remained intact and rigid.	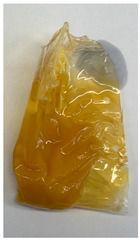 A significant leakage spot was observed, and the film structure was destroyed, losing its rigidity.	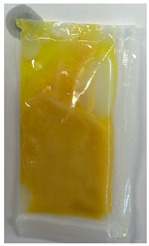 A small puncture hole indicating leakage was detected, and both water and ethanol solutions were able to penetrate inside the pouch during the treatment, but the film structure remained rigid.

## Data Availability

The data presented in this study is available in this article.

## Supplementary Materials

The following supporting information can be downloaded at: https://www.mdpi.com/article/10.3390/polym16050712/s1, Table S1: The recommended solution for optimized condition of pectin/PLA bilayer film; Table S2: The acceptable range and validation results for optimized pectin/PLA bilayer film; Table S3: Light transmission properties of optimized bilayer film as comparison to single film layer; Table S4: Biodegradability rate of optimized bilayer film as comparison to single film layer over 2 weeks period
