# Supplementary material for: Tailoring Pectin-PLA Bilayer Film for Optimal Properties as a Food Pouch Material
Source: Polymers (Basel). 2024 Mar 5;16(5):712. doi: 10.3390/polym16050712 (PMC10933983; doi:10.3390/polym16050712)
Supplement: Supplementary file 1 [file polymers-16-00712-s001.zip › polymers-2877809-supplementary.pdf]

Table S1. The recommended solution for optimized condition of pectin/PLA bilayer film

Number	PLA (g, w/v)	Pectin (g, w/v)	Desirability
1	2.90	1.96	0.85 Selected

Table S2. The acceptable range and validation results for optimized pectin/PLA bilayer film

Test	Predictions	95% CI		95% PI		Validation
		Low	High	Low	High	Experimental value
**TS (mPa)**	6.76	6.31	7.22	5.57	7.96	7.038 ± 0.69
**EAB (%)**	428.38	402.52	454.24	360.94	495.82	462.633 ± 137.95
**WVP (g/msPa)**	1.55×10^{-10}	1.27×10^{-10}	1.82×10^{-10}	8.34×10^{-11}	2.26×10^{-10}	$1.45 \times 10^{-10} \pm 4.58 \times 10^{-11}$
**OP (g/ms)**	9.62×10^{-8}	6.65×10^{-8}	1.26×10^{-7}	1.87×10^{-8}	1.74×10^{-7}	$2.79 \times 10^{-7} \pm 9.20 \times 10^{-8}$
**Solubility (%)**	26.83	24.58	29.06	20.99	32.65	23.53 ± 5.20

* CI = confidence interval; PI = prediction interval; TS = Tensile strength; EAB = Elongation at break; WVP = Water vapor permeability, OP = Oxygen permeability

Table S3. Light transmission properties of optimized bilayer film as comparison to single film layer

Wavelength (nm)	200	280	350	400	500	600	700	800	Opacity (A.mm^{-1})
**Optimized pectin/PLA**	0.38 ± 0.02^{a}	1.66 ± 0.03^{a}	3.18 ± 0.03^{a}	1.53 ± 0.22^{a}	0.89 ± 0.13^{a}	1.22 ± 0.29^{a}	0.84 ± 0.06^{a}	0.88 ± 0.01^{a}	21.08 ± 5.51^{a}
**Pectin**	0.43 ± 0.05^{a}	1.47 ± 0.01^{b}	2.36 ± 0.12^{b}	0.73 ± 0.04^{c}	0.41 ± 0.01^{b}	0.27 ± 0.00^{b}	0.23 ± 0.01^{b}	0.24 ± 0.01^{c}	3.84 ± 0.03^{b}
**PLA**	0.22 ± 0.06^{b}	0.77 ± 0.02^{c}	1.51 ± 0.02^{c}	1.16 ± 0.00^{b}	0.77 ± 0.00^{a}	0.42 ± 0.00^{b}	0.23 ± 0.01^{b}	0.34 ± 0.00^{b}	1.82 ± 0.07^{b}

Table S4. Biodegradability rate of optimized bilayer film as comparison to single film layer over 2 weeks period

**Biodegradation rate (%)**	**0 day**	**3 days**	**7 days**	**14 days**
Optimized pectin/PLA	0%	38.69 ± 1.48%	68.69 ± 5.76%	71.32 ± 4.26%
	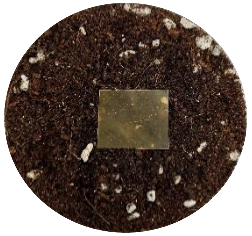	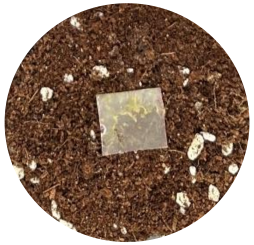	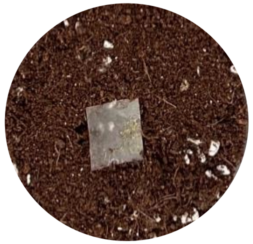	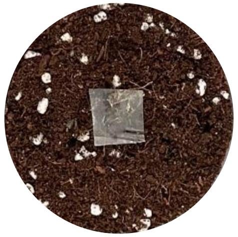
Pectin	0%	100.00 ± 0.00%	100.00 ± 0.00%	100.00 ± 0.00%
	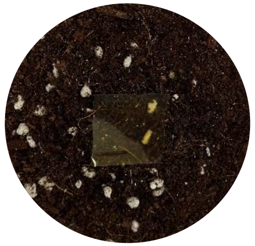	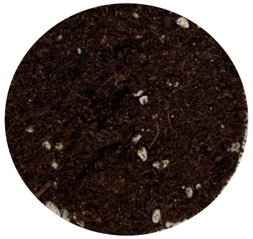	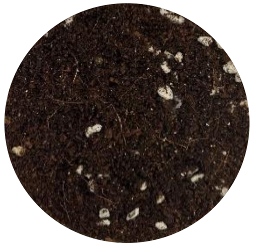	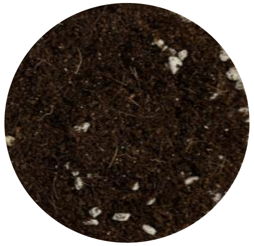
PLA	0%	1.89 ± 1.28%	3.36 ± 2.79%	6.82 ± 0.87%
	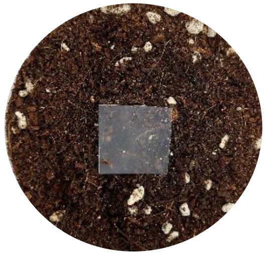	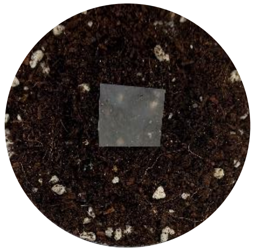	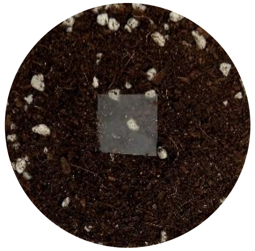	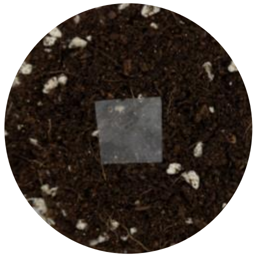
